# Reverse Genetics for Peste des Petits Ruminants Virus: Current Status and Lessons to Learn from Other Non-segmented Negative-Sense RNA Viruses

**DOI:** 10.1007/s12250-018-0066-6

**Published:** 2018-11-19

**Authors:** Alfred Niyokwishimira, Yongxi Dou, Bang Qian, Prajapati Meera, Zhidong Zhang

**Affiliations:** 1 State Key Laboratory of Veterinary Etiological Biology, Lanzhou Veterinary Research Institute, Chinese Academy of Agricultural Sciences, Lanzhou, 730046 China; 2 CAAS-ILRI Joint Laboratory for Ruminant Disease Control, Lanzhou Veterinary Research Institute, Chinese Academy of Agricultural Sciences, Lanzhou, 730046 China

**Keywords:** Peste des petits ruminants (PPR), PPRV, Reverse genetics, Non-segmented negative-sense RNA virus

## Abstract

Peste des petits ruminants (PPR) is a highly contagious transboundary animal disease with a severe socio-economic impact on the livestock industry, particularly in poor countries where it is endemic. Full understanding of PPR virus (PPRV) pathobiology and molecular biology is critical for effective control and eradication of the disease. To achieve these goals, establishment of stable reverse genetics systems for PPRV would play a key role. Unfortunately, this powerful technology remains less accessible and poorly documented for PPRV. In this review, we discussed the current status of PPRV reverse genetics as well as the recent innovations and advances in the reverse genetics of other non-segmented negative-sense RNA viruses that could be applicable to PPRV. These strategies may contribute to the improvement of existing techniques and/or the development of new reverse genetics systems for PPRV.

## Introduction

Peste des petits ruminants virus (PPRV), a member of genus *Morbillivirus* in the family *Paramyxoviridae* (Amarasinghe *et al.*^2018^), is the causative agent of peste des petits ruminants (PPR; Gibbs *et al.*^1979^). PPR is a highly contagious disease of both domestic and wild small ruminants characterized by fever, pneumonia, diarrhea, and inflammation of the respiratory and digestive tracts (Obi *et al.*^1983^; Aruni *et al.*^1998^). Morbidity and mortality rates of the disease can reach up to 100% in susceptible animals, resulting in significant economic losses in endemic countries (Lefevre and Diallo 1990; Sen *et al.*^2010^; Jones *et al.*^2016^). PPRV genome is organized into eight genes in the order 3′-N-P/C/V-M-F-HN-L-5′, which encode six structural proteins and two non-structural proteins (Diallo 1990; Bailey *et al.*^2005^; Baron 2015). The PPRV genome is 15,948 nucleotides (nts) long, which was considered the longest among morbilliviruses until the recent description of the novel *Feline morbillivirus*, whose genome was revealed to be 16,050 nts long (Woo *et al.*^2012^; Marcacci *et al.*^2016^). Like other morbilliviruses, the PPRV genome respects hexamer length and the “rule of six” but has shown a certain degree of flexibility by adding one (+ 1) to two (+ 2) or removing one (− 1) extra nucleotide in mutant minigenomes (Bailey *et al.*^2007^). This unique feature of PPRV is different from other morbilliviruses that strictly obey the “rule of six,” such as the Nipah virus (Halpin *et al.*^2004^). Recently, improvements in molecular techniques have contributed to the molecular understanding of the PPRV genome, although it is still in its infancy (Munir *et al.*^2013^). Furthermore, based on the high homology of PPRV with other morbilliviruses, such as measles virus (MV), canine distemper virus (CDV), and rinderpest virus (RPV; Yoneda *et al.*^2004^; de Vries *et al.*^2012^; Nikolin *et al.*^2012^), at the structural, genetic, and molecular level, much can be deduced about the life cycle of PPRV and its interaction with host cells (Munir *et al.*^2013^); however, the factors that restrict the host range of susceptible animals remain to be investigated (Baron 2015; Baron *et al.*^2016^).

The availability of complete genome sequences from vaccine strains and field isolates for all four lineages of PPRV (Bailey *et al.*^2005^; Muniraju *et al.*^2013^; Dundon *et al.*^2014^) as well as the recent successful implementation of PPRV reverse genetics (Hu *et al.*^2012^; Muniraju *et al.*^2015^) are expected to further enhance our understanding of PPRV. Establishment of more stable reverse genetic systems for PPRV remains a critical tool for understanding the nature of the virus and virus/host interactions. This technique can help elucidate the life cycle of the virus, roles of host and non-host factors in viral replication, pathogenesis, and virulence. Furthermore, reverse genetics is also a powerful tool for developing differentiating infected from vaccinated animals (DIVA) vaccines and companion diagnostic tests that are still lacking for PPRV. The aim of this review is to discuss the current status of PPRV reverse genetics and to provide an extensive overview of recent innovations and advances in reverse genetics of other non-segmented negative-sense RNA viruses. These advances may contribute to the improvement and/or development of reverse genetics techniques for PPRV.

## The Concept and Evolution of Reverse Genetics

In virology, reverse genetics simply refers to the generation of an infectious virus entirely from its complementary DNA (cDNA; Neumann and Kawaoka 2004). In contrast to traditional genetics (or forward genetics), which is based on observing the genetic basis of a phenotype or a trait, reverse genetics moves in an inverse way by analyzing the phenotypic results of specifically engineered genetic sequences (Peters *et al.*^2003^; Hardy *et al.*^2010^; Taniguchi and Komoto 2012). Following advances in molecular biology, reverse genetics was used in the mid-19^th^ century to allow the first rescue of an infectious T2 bacteriophage (Fraser *et al*. 1957). This was then applied to DNA viruses as the DNA could be introduced directly into cells to generate an infectious virus (Pekosz *et al.*^1999^; Armesto *et al.*^2012^). Under the efforts of different researchers, reverse genetics moved to an important stage two decades later with the successful rescue of positive-sense RNA viruses—the bacteriophage Qbeta (Taniguchi *et al.*^1978^) and the mammalian poliovirus (Racaniello and Baltimore 1981). Later, Boyer and Haenni (1994) discovered that *in vitro* transcription of RNA before transfection was more efficient, which was then applied to several RNA viruses. Although virus rescue was seen as a solution to understanding gene function and generating modified viruses to develop new vaccines and virus-based vectors, it was initially restricted to DNA and positive-sense RNA viruses for which *in vitro* synthesized genomic RNA is infectious when transfected to permissive cells (Conzelmann and Meyers 1996).

Negative-sense RNA viruses proved difficult for establishing reverse genetics because genomic RNA alone is not the biological entity for replication, transcription, and translation (Walpita and Flick 2005; Armesto *et al.*^2012^). Furthermore, the naked RNA genome of a negative-sense RNA virus is not infectious and its natural state cannot be found in the cytoplasm. The genome is initially encapsidated by nucleoproteins, forming a ribonucleoprotein (RNP) complex where the antigenome is encapsidated by the positive (+)RNP and serves as a replicative intermediate functioning template for the generation of negative (−)RNP progeny. This is followed by interactions with matrix and viral glycoproteins to create the final budding of a new virion (Armesto *et al.*^2012^; Lamb and Parks 2013). To overcome this problem, different approaches based on the rescue of negative-sense RNA viruses were developed by reconstituting the viral RNP complex. However, these approaches were only successful for segmented viruses such as influenza virus A (Luytjes *et al.*^1989^). The idea of transfecting cells with cDNA encoding the antigenome, rather than the genome, has revolutionized the reverse genetics of non-segmented negative-sense RNA viruses, leading to the successful rescue of rhabdovirus rabies virus (RV; Schnell *et al.*^1994^). However, this approach was not applicable to segmented viruses, which require co-transfection with constructs for each segment and involves modification of the single RNA (Luytjes *et al.*^1989^; Enami *et al.*^1990^). By co-transfecting with constructs for each of the segments, a segmented negative-sense RNA virus, the Bunyamwera bunyavirus was first rescued and was followed by influenza A viruses (Bridgen and Elliott 1996; Fodor *et al.*^1999^; Neumann and Kawaoka 1999). However, rescue of double-stranded RNA viruses remained questionable as these viruses contain a genomic structure that does not naturally occur in cells. Nevertheless, a synthetic transcript of a double-stranded RNA virus was rescued and shown to be infectious (Mundt and Vakharia 1996).

Traditional reverse genetics for each category of viruses have been comprehensively reviewed elsewhere (Nagai 1999; Armesto *et al.*^2012^; Pfaller *et al.*^2015^). Likewise, the general applications of traditional reverse genetics of negative-sense RNA viruses have also been reviewed extensively (Radecke and Billeter 1997; Walpita and Flick 2005; Armesto *et al.*^2012^; Pfaller *et al.*^2015^). Therefore, our review will focus on recent innovations in the rescue of recombinant negative-sense RNA viruses that could be applicable to PPRV.

## Current Status of PPRV Reverse Genetics

Recent advances in molecular biology have contributed to the understanding of PPRV pathobiology and molecular biology. However, there are several gaps that require establishment of stable reverse genetics for a thorough understanding of PPRV, which can contribute to the sustainable eradication of PPR. In addition to improving our current understanding of the nature of the virus, reverse genetics-based studies can accurately provide comprehensive molecular mechanisms of immune induction and can determine the viral proteins involved in immunosuppression during early infection with PPRV. Moreover, reverse genetics is an excellent tool for investigating interactions between viruses and cellular receptors. Using other techniques, it was revealed that short interfering RNA (siRNA)-mediated suppression of signaling lymphocytic activation molecule (SLAM) receptor lead to reduced PPRV titers (Pawar *et al.*^2008^). Overexpression of ovine nectin-4 protein in epithelial cells permitted efficient replication of PPRV, confirming nectin-4 as a PPRV receptor (Birch *et al.*^2013^; Fakri *et al.*^2016^). However, it is believed that more receptors for PPRV exist and that reverse genetics techniques can help discover such new receptors. Furthermore, the availability of a stable reverse genetics system can support the development of DIVA vaccines and companion diagnostic tests that are important for the eradication process and post-eradication screening of the virus. Additionally, application of reverse genetics can lead to the establishment of a PPRV virus vector, as several studies have suggested that recombinant paramyxoviruses are genetically stable vectors due to their relatively simple reverse genetics systems (Walsh *et al.*^2000^; Ge *et al.*^2011^).

Unfortunately, PPRV reverse genetics is not well established. There have been many efforts to rescue PPRV and construct recombinant PPRV, which can be engineered, but the related literature remains insufficient and the main cause of failure is not fully or well documented. An early report in 2007 attempted to develop a reverse genetics system for PPRV but was unsuccessful (Bailey *et al.*^2007^). Although this established system was only verified at the minigenome level, it was demonstrated that PPRV rescue elements include the antisense PPRV cDNA, PPRV genome promoter (GP), and PPRV antigenome promoter (AGP) flanked by hepatitis delta virus ribozyme (HDVRZ). In a comparison study of the PPRV heterologous and homologous helper plasmids with a previously established reverse genetics system for RPV, the PPRV homologous helper plasmids performed well in minigenome rescue, whereas expression in transfected cells indicated that PPRV did not strictly obey the “rule of six” in contrast with other paramyxoviruses (Bailey *et al.*^2007^). Biological activity of the PPRV polymerase gene (*L*) was previously analyzed and the role of RNA-dependent RNA polymerase (*RdRp*) was determined in attempted reverse genetics involving the N, P, and L proteins as well as the PPRV leader and trailer for minigenome expression (Minet *et al.*^2009^). Construction of the full-length cDNA clone was also reported in an attempted PPRV rescue assay (Zhai *et al.*^2010^) with no evidence of virus rescue.

Despite the hypothesis that the high GC-rich region of the PPRV genome (between the open reading frame of *M* and *F* genes) is a potential bottleneck for viable PPRV rescue (Bailey *et al.*^2007^), neither evidence of virus rescue nor a cause for failure was further reported or discussed. It was not until 2012 that a GFP-expressing recombinant PPRV was rescued from a PPRV full-length cDNA clone (Hu *et al.*^2012^). The second and last known successful PPRV rescue was based on a commercially synthesized plasmid containing the full-length PPRV antigenome sequence with an inserted enhanced GFP (eGFP) sequence (Muniraju *et al.*^2015^). In these two successful PPRV rescue systems, the rescued viruses were assessed for application in rapid virus neutralization tests (Hu *et al.*^2012^) in comparison with the standard vaccine strain. A DIVA system assay with the rescued, positively marked recombinant virus through eGFP insertion and the negatively marked recombinant virus through mutation of the C77 monoclonal antibody binding epitope on the PPRV H gene was also assessed (Muniraju *et al.*^2015^). Although these two available reverse genetics systems for PPRV can serve as good references, information regarding further application is still lacking. Therefore, there is still a need to establish or improve existing systems to efficiently understand the biology and pathogenicity of the virus and contribute to the planned PPRV eradication program.

## Recent Innovations in Reverse Genetics of Other Non-Segmented Negative-Sense RNA Viruses

PPRV belongs to the family *Paramyxoviridae* in the order *Mononegavirales*, which also includes *Rhabdoviridae, Nyamaviridae, Bornaviridae, Filoviridae*, and *Pneumoviridae* (Amarasinghe *et al.*^2018^). These viruses share a common feature in reverse genetics—their RNA is not an infectious unit before they are packaged by nucleoproteins and transcribed by polymerase and other required co-factors. The history of successful reverse genetics for these virus families dates from the first rescue of an RV (Schnell *et al.*^1994^). The possibility of virus engineering by nucleotide insertion or deletion at will has revolutionized our molecular understanding of these viruses. In the following sections we will present an overview of recent innovations in design and new strategies that can serve as references for improving or establishing PPRV reverse genetic systems.

### (a) Initial RNA Transcription and Cleavage at the 5′ and 3′-ends

The success of reverse genetics for non-segmented negative-sense RNA viruses is influenced by several factors, including the intact full-length cDNA of the virus to be rescued and its correct 5′ and 3′-ends. T7 RNA polymerase activity tends to initiate from error-free templates at both 5′ and 3′-ends of an RNA transcript. The so-called leader and trailer regions play a critical role in RNA transcription and virus replication (Yunus *et al.*^1999^; Hanley *et al.*^2010^) and thus these regions must be kept intact. In all reverse genetics systems analyzed thus far, mutations in both the leader and trailer sequences have shown a negative influence on RNA transcription and virus replication (Peeples and Collins 2000; Hanley *et al.*^2010^). To avoid extraneous nucleotides from inserting in the 5′ and 3′-ends of the RNA template during *in vitro* transcription, several methods have been described for yielding target RNAs with precise and defined ends (Pleiss *et al.*^1998^; Helm *et al.*^1999^; Kao *et al.*^1999^; Avis *et al.*^2012^). It is now believed that the possible heterologous 5′ and 3′-ends during run-off transcription with T7 RNA polymerase can be controlled by self-cleaving trans and cis-acting ribozymes. Thus a hammerhead ribozyme (HHRZ) at the 5′-end and HDVRZ at the 3′-end have been introduced and are widely used with high efficiency (Been and Wickham 1997; Wichlacz *et al.*^2004^; Avis *et al.*^2012^; Szafraniec *et al.*^2012^; Meyer and Masquida 2014). However, there is no common standard design applicable for all viruses and thus each system must be adapted and optimized for each particular virus. For example, insertion of HHRZ between the T7 promoter and start codon of the minigenome significantly improved rescue efficiency of the RV minigenome. This approach increased rescue efficiency by 100-fold for a full-length RV in combination with HDVRZ flanking the 3′-end of the antigenome (Ghanem *et al.*^2012^). Surprisingly, the same approach showed poor performance with MV or Borna disease virus (BDV; Martin *et al.*^2006^). Moreover, HHRZ sequences are obtained from different families of endonucleolytic ribozymes and may possess variations in cleavage efficiency among different sequences (Hammann *et al.*^2012^).

With continued improvements in molecular biology, reverse genetics technology has progressed within the last two decades. In traditional reverse genetics, T7 RNA polymerase was widely used for negative-sense RNA viruses for which the majority of RNA transcription is accomplished in the cytoplasm (Edenborough and Marsh 2014). To improve the reverse genetics of these viruses, other alternative systems were developed, such as replacement of the T7 promoter by the human cytomegalovirus promoter (CMV), which is directly recognized by eukaryotic RNA polymerase (Inoue *et al.*^2003^; Martin *et al.*^2006^; Wang *et al.*^2015^; Liu *et al.*2017a). Following the same approach, Hu *et al.* (2012) developed a CMV promoter-based system and successfully rescued PPRV from full-length PPRV cDNA for the first time. In previous attempts, a similar approach was applied to improve RV recovery (Inoue *et al.*^2003^). Although the T7 promoter-based system has been widely used to rescue negative-sense RNA viruses, this system requires the use of an exogenous T7 RNA polymerase—usually from the vaccinia virus (vTF7-3)—which interferes with the viability of transfected cells (Nakatsu *et al.*^2006^). This problem was overcome by adding vaccinia virus replication inhibitors, such as cytosine arabinoside and rifampicin, which increased the viability of transfected cells (Kato *et al.*^1996^). Additionally, using mutant vaccinia virus (MVA-T7) that grows in avian but not mammalian cells also avoided the vaccinia-induced cytotoxicity (Sutter *et al.*^1995^; Wyatt *et al.*^1995^). Efforts to develop helper virus-free systems with transgenic cell lines expressing T7 RNA polymerase have also been successful (Martin *et al.*^2006^; Zheng *et al.*^2009^; Li *et al.*^2011^). Furthermore, the use of T7 RNA polymerase-expressing plasmids prior to or during co-transfection with the full-length antigenome and helper plasmids for generating infectious viruses has also been reported (Lowen *et al.*^2004^; Witko *et al.*^2006^; Freiberg *et al.*^2008^; Jiang *et al.*^2009^). Detailed information on the different systems used to rescue negative-sense RNA viruses in traditional reverse genetics have been extensively reviewed elsewhere (Radecke and Billeter 1997; Huemer *et al.*^2000^; Edenborough and Marsh 2014).

There have been increasing reports of successful negative-sense RNA virus rescue using cellular inherent RNA polymerase I (Pol I) (Murakami *et al.*^2008^; Suphaphiphat *et al.*^2010^) and RNA polymerase II (Pol II)-driven systems under the control of a CMV promoter (Li *et al.*^2011^; Wang *et al.*^2015^). It was initially thought that the possibility of BDV rescue with RNA Pol I and RNA Pol II was due to its unique genetics and biological features of being the only member of *Mononegavirales* to exhibit nuclear replication (de la Torre 2002; Perez *et al.*^2003^; Lipkin *et al.*^2011^). A CMV promoter-driven RNA Pol II system was first thought to function better with nuclear-replicating viruses; however, this hypothesis does not correlate with the recent rescue of other non-nuclear replicating viruses, such as PPRV and Newcastle disease virus (NDV), using the same promoter (Hu *et al.*^2012^; Wang *et al.*^2015^; Liu *et al.*2017a). Other exceptions to this hypothesis include influenza viruses, MV, and Ebola virus. These cytoplasmic-based RNA transcription viruses have been rescued by cellular RNA Pol I or RNA Pol II (Edenborough and Marsh 2014). Despite these findings, further investigations are still needed on the utility of Pol II for virus rescue of other negative-sense RNA viruses due to the possible splicing or polyadenylation of Pol II transcripts (Martin *et al.*^2006^).

The above examples leave room for hypothesizing alternative methods that can be explored for virus rescue of other non-nuclear transcription viruses including PPRV. Of note, the Pol I and Pol II-driven systems—under the control of a CMV promoter—can avoid helper virus-induced cytopathic effects (CPE) after cell transfection, which may be confused with the CPE induced by the rescued virus. Indeed, the CMV promoter was successfully used to rescue NDV, although it was shown to be less efficient in low virulent strains (Liu *et al.*2017a). This low efficiency in rescuing low virulent strains was previously linked with system complexity involving several different-sized plasmids and the poor capacity of low virulent strains to be rescued, as described in the past for segmented influenza viruses (Neumann *et al.*^2005^). In this regard, considering that all available methods for PPRV rescue employ the PPRV Nigeria75/1 strain (an attenuated vaccine strain) as template, we propose that a comparison of rescue efficiency of PPRV Nigeria75/1 strain with other virulent PPRV strains is necessary to assess possible limitations of virus rescue, which may be linked with low virulence of PPRV Nigeria75/1. However, due to biosecurity concerns, such comparative studies may require specialized laboratories that are licensed to handle live virulent PPRV.

### (b) Generation of an Intact Full-length cDNA

The *Paramyxoviridae* family includes enveloped viruses with linear non-segmented negative-sense RNAs that are approximately 15.5 kb in length. Their active polymerase is usually a complex of at least two components and the initiation of the viral cycle is a complex of virion-associated RdRp that generates a functional RNP complex. Although molecular techniques such as PCR, gene cloning, and the use of endonuclease restriction enzymes have become routine, generation of an intact, error-free, and stable clone of more than 15 kb is not always easy. The PPRV genome (15,948 nts) is one of the longest paramyxoviruses after the recently described novel *Feline morbillivirus* (16,050 nts; Woo *et al.*^2012^; Marcacci *et al.*^2016^). Such large genome sizes are relatively difficult for generating error-free, full-length cDNA using conventional cloning techniques. To overcome these potential sequence errors, a synthetic approach was applied to generate error-free cDNA in a PPRV rescue assay (Muniraju *et al.*^2015^). However, this approach is not 100% error-free in cases where wild-type strains are to be rescued due to possible errors that may exist in the sequences available in GenBank. These limitations linked to genome size and the ability to generate stable full-length cDNA plasmids were previously reported during an attempt at establishing a one plasmid-based system to rescue NDV. The 33 kb pMG-725/GFP-NPL plasmid was unstable due to its size and was lost during passaging in new bacterial culture medium (Liu *et al.*2017b).

Alternative methods for generating long template cDNAs, such as the faster and more economic methods used in clone screening (Guo *et al.*^2007^), should thus be considered. Recently, fast screening of the clones after transformation was shown to be more advantageous compared with conventional restriction and PCR methods (Liu *et al.*2017b). Similar innovations in facilitating long DNA cloning, such as enzyme-free cloning and the recently modified enzyme-free cloning protocols, are continuously being developed (Tillett and Neilan 1999; Blanusa *et al.*^2010^; Matsumoto and Itoh 2011). A new strategy for rapid generation of complete cDNA clones of negative-sense RNA and recombinant viruses (Nolden *et al.*^2016^) is based on direct cloning of cDNA copies of a complete virus genome into reverse genetics vectors through a technique called “linear-to-linear RedE/T” recombination. This convenient technique has been long used to manipulate molecules such as yeast, bacteria, P1-derived artificial chromosome vectors, and *Escherichia coli* chromosomes (Zhang *et al.*^1998^). Techniques associated with this method have been shown to be appropriate for direct cloning of long DNA sequences (Fu *et al.*^2012^; Wang *et al.*^2016^) and may constitute alternative methods of reducing the usual conflicts between restriction sites on plasmid vectors and gene inserts. In addition, these alternative methods reduce errors when compared with long-term manipulations of genome sequences under conventional techniques.

### (c) Cell Lines for Virus Rescue

In virology, virus isolation is the core of any advancement in research. Isolation of a virus usually requires sensitive cells that allow viral growth and replication. Moreover, virus rescue from cDNA requires highly sensitive and permissive cell lines to allow effective replication and propagation of a rescued virus. In laboratory settings, several viruses of grazing animals, such as PPRV, sheep and goat pox virus (SPV), and Orf virus, usually exhibit poor growth *in vitro* and show difficulty adapting to commonly used cell lines or animal models (personal communication). Consequently, compared with other morbilliviruses, isolation of a field PPRV strain can be difficult due to the lack of sensitive cell lines or inadequate conditions of transportation and stocking of samples (Bhuiyan *et al.*^2014^), especially in poor endemic countries. In this section, we will explore the available options for PPRV growth and replication in different cell lines to aid appropriate cell line choice for virus rescue assays.

Conventional cell lines that exhibit high performance in growth and propagation of PPRV are rarely available (Fakri *et al.*^2016^). Fortunately, there is an increasing number of reports of engineered cell lines expressing known receptors such as SLAM and nectin-4 for other morbilliviruses that have been shown to be or may be more sensitive to supporting PPRV growth and replication (Hsu *et al.*^2001^; Adombi *et al.*^2011^; Muhlebach *et al.*^2011^; Noyce and Richardson 2012; Noyce *et al.*^2013^). In addition, a lymph node suspension cell line derived from cow showed higher sensitivity to PPRV in comparison with adherent Vero cells. The high titer of PPRV in the cow-derived lymph node cell line was linked to the fact that lymphoid cells are major targets of different morbilliviruses (Mofrad *et al.*^2016^). In a comparative study of potential permissive cell lines for PPRV growth and propagation, both BHK-21A and HEK 293T cells were able to produce PPRV titers (Silva *et al*. 2008). Research results have led to different opinions on the growth and replication of PPRV in various cell lines (Seki *et al.*^2003^; Emikpe *et al.*^2009^; Sannat *et al.*^2014^; Muniraju *et al.*^2015^; Fakri *et al.*^2016^; Latif *et al.*^2018^). Therefore, it is critical to select a highly sensitive cell line during PPRV rescue assay; engineered cell lines that exhibit high sensitivity for PPRV growth and replication are listed in Table 1.

**Table 1:** Samples of engineered sensitive cell lines for PPRV growth and replication.

Cell line	Remark	References
Monkey CV1 expressing the sheep-goat SLAM protein (CHS-20)	A highly sensitive cell line for the isolation of PPRV from pathological specimens	Adombi *et al.* (2011)
VeroDogSLAMtag (VDS)	Widely and efficiently used in isolation of different morbilliviruses including PPRV	Seki *et al.* (2003), Muniraju *et al.* (2015)
Vero/SLAM	Vero cell line expressing SLAM receptors is a highly sensitive *in vitro* system for cultivation of PPRV	Sannat *et al.* (2014)
VeroNectin-4	VeroNectin-4 cells are ideal for PPRV isolation from fields samples as well as for the titration of PPRV	Fakri *et al.* (2016)
BTS-34	BST-34 is a CV1-based cell line constitutively expressing the bovine SLAM. In comparison with Vero-76 cell line, BTS-34 produced higher titer of PPRV	Latif *et al.* (2018)

PPRV, Peste des petits ruminants virus; SLAM, signaling lymphocytic activation molecule.

## New Strategies for Virus Rescue

Theoretically, co-transfection of antigenomic cDNA representing the full-length RNA of a non-segmented negative-sense RNA virus with appropriate plasmids expressing RNP in the presence of a suitable source of T7 RNA polymerase will result in recovery of an infectious virus (Schnell *et al.*^1994^; Armesto *et al.*^2012^; Pfaller *et al.*^2015^). In practice however, virus rescue is a complex process influenced by several factors including essential viral replication proteins and the functional viral RNA template. With that in mind, there is no standard protocol for reverse genetics even within the same family of viruses. In an effort to continually improve reverse genetics for PPRV, we will discuss below the recent and novel strategies used for rescue of other negative-sense RNA viruses, which may be successfully applied to PPRV.

**Fig. 1:**
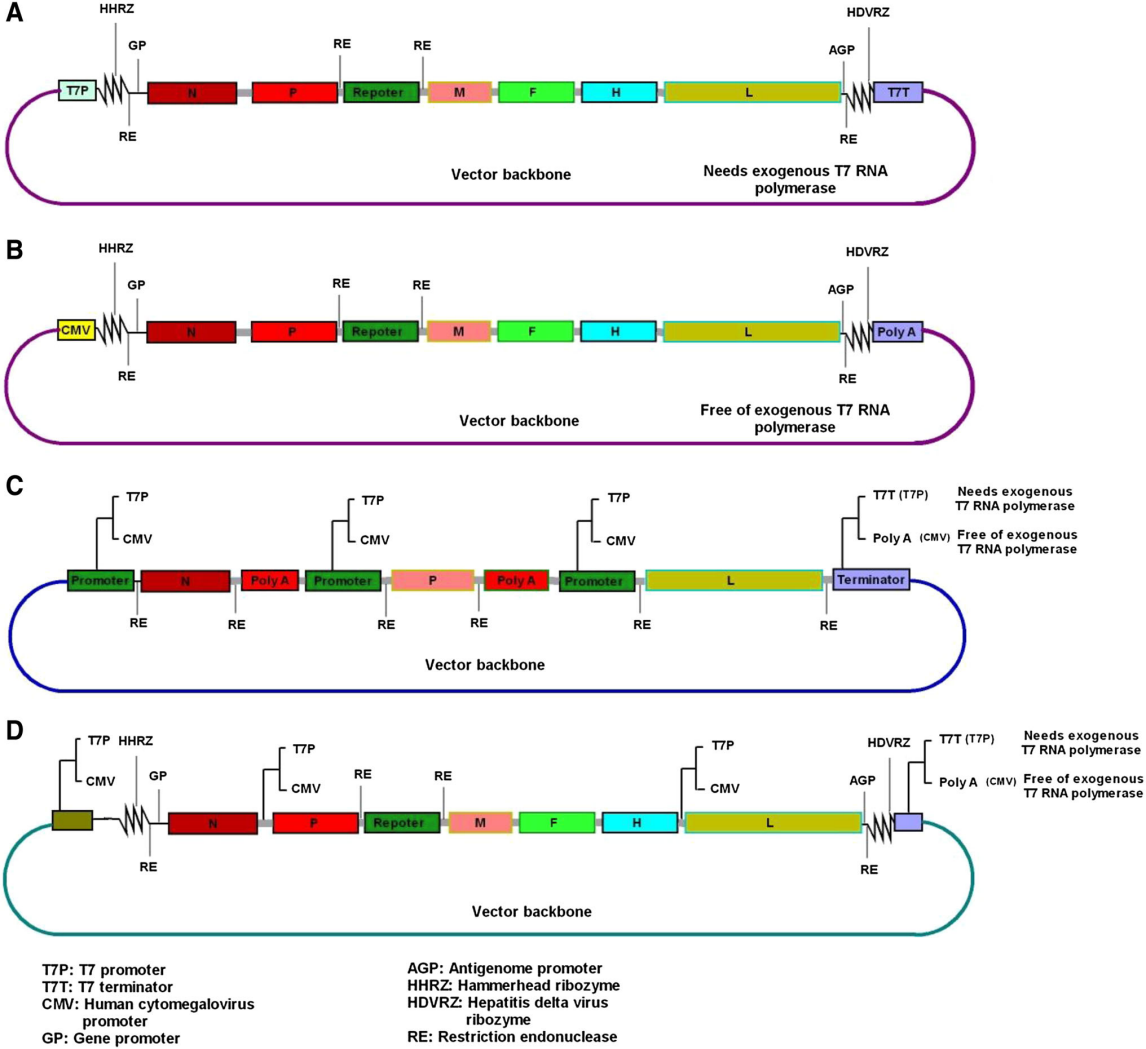
Summary illustrating the recent innovations in experimental design of rescuing recombinant non-segmented negative-sense RNA viruses. **A, B** Design of the full-length cDNA antigenome flanked by trans and cis-acting ribozymes to generate the correct 5′ and 3′-ends in T7 and CMV promoter-driven systems for initial RNA transcription. The strategy in **A** requires exogenous T7 RNA polymerase while **B** is free of exogenous T7 RNA polymerase and relies on the CMV promoter. **C** The two-plasmid system design with a single helper plasmid encoding three translational cassettes of the essential viral replication proteins (N, P, and L). Each translational cassette is spanned with an appropriate promoter (T7 or CMV) and terminator (T7T or poly-A tail) that are dependent on the rescue strategy and with or without exogenous T7 RNA polymerase. In this system, only the plasmid containing the full-length antigenome and the single helper plasmids will be co-transfected to generate an infectious virus. **D** The design of the one plasmid and helper plasmid free based-system that implements both T7 and CMV promoter-driven systems with or without exogenous T7 RNA polymerase. In this system, additional promoter (T7 or CMV) sequences are inserted by careful substitution into the viral cDNA at strategic positions. This allows transcription of sub-genomic RNAs that encode essential viral replication proteins (N, P, and L) that are needed for the RNP complex to form. The T7 promoter-based system requires an exogenous T7 RNA polymerase and the CMV promoter-based system is free of exogenous T7 RNA polymerase.

In traditional reverse genetics of negative-sense RNA viruses (mostly with cytoplasmic RNA transcription), virus rescue relies on co-transfection into eukaryotic cells with at least four plasmids representing the full-length antigenomic sequence of the virus and helper plasmids (N, P, and L) independently cloned downstream of the T7 promoter in the presence of an exogenous T7 RNA polymerase source. Even though rescue efficiency for some viruses with cytoplasmic replication can be improved under the control of Pol I and Pol II, the T7 promoter is being gradually replaced by the CMV promoter, which is directly recognized by eukaryotic RNA polymerase (Inoue *et al.*^2003^; Martin *et al.*^2006^; Wang *et al.*^2015^; Liu *et al.*2017a). This replacement has the advantage of avoiding exogenous T7 RNA polymerase, which reduces system performance due to CPE caused by the helper virus or the increased number of co-transfected plasmids. On the other hand, the ability of codon-optimized T7 polymerase to drive paramyxovirus rescue was reported to be robust and has increased the efficiency of virus rescue for major paramyxoviruses (Beaty *et al.*^2017^). In most traditional reverse genetics, HDVRZ was widely used downstream of the antigenome sequence to generate correct 3′-ends. However, an improved design with trans and cis-acting ribozymes (HHRZ and HDVRZ) flanking the antigenome upstream and downstream, respectively (as shown in Figs. 1A, 1B), has shown a more efficient performance. Thus, cDNA clones flanked by a combination of optimized 3′ and 5′-ribozymes upstream and downstream to generate the exact 3′ and 5′-ends increased RV rescue by at least 100-fold (Ghanem *et al.*^2012^). Furthermore, a reduced number of plasmids co-transfected during virus rescue increased rescue efficiency in segmented viruses (Neumann *et al.*^2005^; Zhang and Curtiss 2015), double-stranded RNA viruses (Kobayashi *et al.*^2010^), and Nipah virus (Yun *et al.*^2015^). Another important aspect to consider in reverse genetics is the design of appropriate transcription termination elements, which are still a point of discussion in molecular biology (Richard and Manley 2009; Porrua and Libri 2015).

In view of the abovementioned points, we will further discuss design and rescue strategies that could be applied to improve existing systems or develop new reverse genetics systems for PPRV (Figs. 1C, 1D). These designs are either T7 or CMV promoter-driven systems depending on the facilities available in the laboratory.

### (a) Two-Plasmid-Based System

The two-plasmid system is a new approach consisting of a single helper plasmid encoding three translational cassettes of essential viral replication proteins (N, P, and L) cloned into one plasmid vector as illustrated in Fig. 1C, which was previously described by Liu *et al.* (2017a, b). In this system, each translational cassette is spanned with an appropriate promoter (T7 or CMV) and terminator (T7T or poly-A tail) and—depending on the rescue strategy—with or without exogenous T7 RNA polymerase. In this system, only a plasmid containing the full-length antigenome of the virus and a single helper plasmid will be co-transfected into a eukaryotic cell to generate an infectious virus. Compared with the traditional four-plasmid system, the two-plasmid system exhibited a 100% rescue efficiency against the 67% seen for the four-plasmid system and had higher (4.5-fold) NDV virus titers. Moreover, the two-plasmid system was found more efficient in the rescue of lentogenic viruses and can rescue viruses that were not possible by the four-plasmid system (Liu *et al.*2017a, b).

### (b) Single-Plasmid-Based System

The single-plasmid system is a helper plasmid-free-based system that may be driven by a T7 or CMV promoter with or without exogenous T7 RNA polymerase (Fig. 1D). In this system, additional promoter (T7 or CMV) sequences are inserted by careful substitution in the viral cDNA at strategic positions. This allows independent transcription of sub-genomic RNAs encoding essential viral replication proteins (N, P, and L) that allow the RNP complex to form. Although this technique showed less efficiency in virus replication compared with that of the parental virus, it could rescue NDV by using a single full-length viral cDNA plasmid that included T7 promoter sequences at strategic positions (Peeters and de Leeuw 2017). Due to similarities in replication strategies, the single-plasmid system is predicted to be applicable to most non-segmented negative-sense RNA viruses. Theoretically, it may work with or without exogenous T7 RNA polymerase, depending on the promoter (T7 or CMV) that triggers the initial RNA transcription. Not only has this method worked for NDV, but it also showed the advantages of having a reduced size with promoter sequences inserted by sequence substitution into the intergenic untranslated regions (UTRs) of sub-genomic RNAs encoding essential viral replication proteins. However, it is critical to carefully select the substitution region to avoid disturbing essential elements that enhance the expression of downstream genes in the viral genome. Previously, a similar approach was assayed by the construction of a single plasmid that included four translational cassettes representing the full-length sequence of the NDV and the three helper plasmids representing the NDV N, P, and L proteins (Liu *et al.*2017a). However, this construct was unsuccessful, and the author suspected the large size (33 kb) of the plasmid as the reason for failure.

## Conclusion

Years after the approval of a global strategy for the control and eradication of PPRV, there are still continued reports of new PPRV cases, even in unusual hosts, worldwide (Boussini *et al.*^2016^; Marashi *et al.*^2017^; Shatar *et al.*^2017^). Until now, research on several areas of basic and applied virology is still lacking, particularly, with respect to the mechanisms of disease transmission, epidemiology, virus life cycle, and the role of wildlife reservoirs in disease persistence and propagation, which are not yet well defined (Baron *et al.*^2016^, ^2017^). In addition, the pathobiology and molecular biology of PPRV are still not fully understood (Baron 2015; Munir 2015). The next generation of vaccines and diagnostic tests including DIVA systems are also lacking (Munir *et al.*^2013^; Baron *et al.*^2017^). In this review, we discussed the recent advances in reverse genetics technology of non-segmented negative-sense RNA viruses that may be applicable to PPRV. We also proposed several designs that may improve existing strategies or promote the development of new reverse genetic techniques for PPRV. Reverse genetics is a powerful tool that may provide solutions to understanding this economically important pathogen, supporting the ongoing efforts of PPRV sustainable control and eradication.
